# Copeptin Implementation on Stroke Prognosis

**DOI:** 10.3390/neurolint15010008

**Published:** 2023-01-16

**Authors:** Stella Karatzetzou, Dimitrios Tsiptsios, Anastasia Sousanidou, Styliani Fotiadou, Foteini Christidi, Christos Kokkotis, Aimilios Gkantzios, Eleftherios Stefas, Pinelopi Vlotinou, Antonia Kaltsatou, Nikolaos Aggelousis, Konstantinos Vadikolias

**Affiliations:** 1 Neurology Department, Democritus University of Thrace, 68100 Alexandroupolis, Greece; 2 Department of Physical Education and Sport Science, Democritus University of Thrace, 69100 Komotini, Greece; 3 FAME Laboratory, Department of Physical Education and Sport Science, University of Thessaly, 42100 Trikala, Greece

**Keywords:** copeptin, stroke, cerebrovascular disease, prognosis, recovery, blood biomarker

## Abstract

Predicting functional outcome following stroke is considered to be of key importance in an attempt to optimize overall stroke care. Although clinical prognostic tools have been widely implemented, optimal blood biomarkers might be able to yield additional information regarding each stroke survivor’s propensity for recovery. Copeptin seems to have interesting prognostic potential poststroke. The present review aims to explore the prognostic significance of copeptin in stroke patients. Literature research of two databases (MEDLINE and Scopus) was conducted to trace all relevant studies published between 16 February 2012 and 16 February 2022 that focused on the utility of copeptin as a prognostic marker in acute stroke setting. 25 studies have been identified and included in the present review. The predictive ability of copeptin regarding both functional outcome and mortality appears to be in the range of established clinical variables, thus highlighting the added value of copeptin evaluation in stroke management. Apart from acute ischemic stroke, the discriminatory accuracy of the biomarker was also demonstrated among patients with transient ischemic attack, intracerebral hemorrhage, and subarachnoid hemorrhage. Overall, copeptin represents a powerful prognostic tool, the clinical implementation of which is expected to significantly facilitate the individualized management of stroke patients.

## 1. Introduction

Stroke represents not only the second leading cause of death worldwide but also the primary source of acquired disability among adult population [1,2]. When taking both the constantly expanding world’s population and the significantly improving life expectancy as well as the age profile of most stroke patients into account, stroke burden will continue to rise across the world, especially in low-income countries [3]. Thus, a growing need emerges for a timely and accurate prognosis of each patient’s propensity for functional recovery poststroke, as being able to forecast rehabilitation potential following stroke is of great importance in terms of overall stroke management and decision making.

Stroke heterogeneity in terms of etiology and pathophysiology provides important insight into the challenging nature of long-term outcome prediction poststroke [4,5]. Various clinical evaluation scales and neurophysiological techniques have been developed and proved to be of prognostic significance in terms of individualized post-stroke recovery potential [5,6]. Among the established clinical scores with prognostic potential are the National Institutes of Health Stroke Scale (NIHSS) [7] and the ABCD2 score (age, blood pressure, clinical features of transient ischemic attack, duration of symptoms and presence of diabetes mellitus) [8], which are considered able to provide an estimation of ischemic stroke (IS) functional outcome and mortality, as well as the stroke recurrence following a transient ischemic attack (TIA), respectively. As far as the prognostic tools utilized in cases of intracerebral hemorrhage (ICH) and subarachnoid hemorrhage (SAH), there are clinical scales that are widely implemented, including the ICH score [9], the Hunt and Hess scale [10] and the World Federation of Neurological Surgeons (WFNS) scale [11]. However, given the variability of stroke clinical manifestation, clinical information solely has not proved sufficient for accurate prognostication following stroke [12]. Thus, biomarker-based approaches in a setting of acute stroke might serve as an adjunctive tool in an attempt to reliably predict each stroke survivor’s recovery potential [13,14,15].

According to the Biomarkers Definitions Working Group, the term biomarker refers to “a characteristic that is objectively measured and evaluated as an indication of normal biological processes, pathogenic processes, or pharmacologic responses to a therapeutic intervention” [16]. In acute stroke setting, the evaluation of selected blood biomarkers may play a role in establishing the diagnosis, predicting the functional outcome, as well as promoting recovery poststroke. An ideal biomarker should not only be characterized by high sensitivity and specificity but also be reproducibly and noninvasively obtained from easily available sources in an attempt to optimize prognostication of stroke outcome. Furthermore, stroke recovery biomarkers should reflect underlying pathophysiological processes of the ischemic cascade to prove of clinical benefit [13,17,18]. Over the last few years, a steadily increasing understanding of stroke pathophysiology resulted in the identification and development of several blood biomarkers, including peptides/enzymes, inflammatory variables, oxidative/metabolic markers, hematological/vascular indicators, and hormones, such as vasopressin (AVP) and copeptin [19].

The body’s stress response aiming at re-establishing homeostasis after an acute cerebrovascular event includes the activation of the hypothalamic–pituitary–adrenal (HPA) axis. Considered between the first measurable physiological reactions to cerebral ischemia [20], the subsequent hormonal cascade results in releasing several stress mediators, AVP and copeptin being among them. AVP constitutes a key regulator of water balance within brain tissue and is being produced shortly following stroke in equimolar amounts to copeptin. Nevertheless, the evaluation of circulating AVP levels appears challenging, when taking the hormone’s unstable nature and rapid clearance from plasma into account [21,22]. Copeptin, a brain-derived stress hormone that shares the same precursor with AVP, is easy to determine and is characterized by significant molecular stability within circulation, thus emerging as a useful surrogate marker for AVP. Being derived from pro-vasopressin along with AVP, copeptin reflects the activation of the endogenous stress system, exhibiting prognostic potential in stroke patients [23,24]. 

It is noteworthy that copeptin seems able to accurately mirror stroke severity and differentiate between patients with favorable outcomes and patients with poor outcomes. Interestingly, the assessment of plasma copeptin concentration may enhance the prognostic value of already validated clinical scores by independently predicting both stroke functional outcome and mortality [23]. More specifically, elevated copeptin levels at admission were found to be significantly correlated with unfavorable outcomes, as well as carried a higher risk of all-cause death within stroke population [25]. Additionally, copeptin may play an important role in prognostication following TIA, as it provides additional information beyond commonly utilized clinical scoring tools, thus improving the discriminatory accuracy regarding recurrent cerebrovascular events [26]. 

Taking the urgent need for accurate prognosis provided early after stroke and the potential role of copeptin in providing an estimation of each individual’s recovery potential into account, the purpose of the present study was to review all available literature published within the last decade dealing with copeptin as a prognostic indicator not only in cases of IS but also in patients presenting with TIA, ICH and SAH.

## 2. Materials and Methods

The Preferred Reporting Items for Systematic Reviews and Meta-analyses (PRISMA registration number: CRD42022385442) was used to guide this study. Our study’s methods were a priori designed.

### 2.1. Search Strategy 

Literature research of two databases (MEDLINE and Scopus) was conducted by one investigator (SK) in order to trace all relevant studies published between 16 February 2012 and 16 February 2022, using “copeptin” AND [“stroke outcome” OR “stroke prognosis”] as keywords. Also, the terms: (1). “AIS” OR “acute ischemic stroke”, (2). “TIA” OR “transient ischemic attack”, (3). “ICH” OR “intracerebral hemorrhage” and (4). “SAH” OR “subarachnoid hemorrhage” were utilized as secondary search criteria. The retrieved articles were also hand-searched for any further potential eligible articles. Any disagreement regarding the screening, or selection process, was solved by a second investigator (KV) until a consensus was reached.

### 2.2. Selection Criteria

Only full-text original longitudinal studies published in the English language were included. Cross-sectional studies, secondary analyses, reviews, guidelines, meeting summaries, comments, unpublished abstracts, or studies conducted on animals were excluded. 

### 2.3. Data Extraction

Data extraction was performed using a predefined data form created in Excel. We recorded the type of stroke, authors, year of publication, number of participants, mean or median age of participants, time of copeptin measurement, cutoff values (specificity, sensitivity), follow-up time, assessment stroke scales and main results.

### 2.4. Data Analysis

No statistical analysis or meta-analysis was performed due to the high heterogeneity among studies.

Thus, the data were only descriptively analyzed.

## 3. Results 

### 3.1. Database Searches

**Figure 1:**
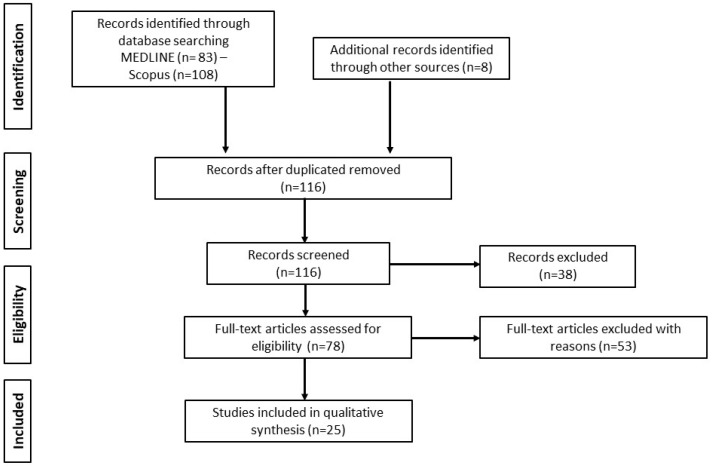
Study flow chart (PRISMA diagram).

199 records were retrieved following database search. 83 duplicates and 38 irrelevant studies were excluded; hence, a total of 78 articles were selected. After screening the full text of the articles, 53 were excluded for reasons mentioned in Section 2.2, resulting in 25 studies being eligible for inclusion (Figure 1).

### 3.2. Study Characteristics

Twenty-five publications fulfilled our inclusion criteria. They were classified into 4 groups, according to the type of stroke investigated. The first group consisted of twelve studies focusing on the prognostic potential of serum copeptin among ischemic stroke patients [27,28,29,30,31,32,33,34,35,36,37,38,39]. The second group included three studies dealing with the predictive ability of circulating copeptin levels following TIA in terms of stroke recurrence, functional outcome and mortality [40,41,42]. The third group comprised of five studies exploring the prognostic significance of copeptin in an acute ICH setting regarding clinical outcome and survival [43,44,45,46,47]. The fourth group embodied four studies examining the role copeptin may play in forecasting the prognosis and potential complications in SAH population [48,49,50,51]. Finally, one study investigated the discriminatory accuracy of copeptin concentration in patients suffering from either ischemic stroke or TIA [39] (Table 1). 

### 3.3. Stroke Patient Groups and Demographic Profile

The total number of stroke patients included in all studies ranges from n = 18 [48] to n = 4125 [34]. Across the 25 studies, 6 studies have a disease sample size between 1–100 patients, 8 studies between 101–200, 6 studies between 201–300 and 5 studies have a disease sample size larger than 300 patients. Mean patients’ age ranges from 45.06 ± 9.78 [47] to 72.1 ± 12.1 [38] years.

### 3.4. Reference Groups

Across the 25 studies, stroke patients are contrasted to demographically matched healthy individuals in 12 studies, with the rest of them not including a healthy control group. None of the studies include a disease-control group other than stroke patients.

### 3.5. Time of Blood Sampling

In 4 studies blood sampling was performed upon admission, in 1 study within 6 h of symptom onset, in 8 studies in the first 24 h, in 6 studies in the first 48 h, in 1 study in the first 72 h, in 2 studies within the first 4 days from admission, in one study copeptin measurement was performed upon admission and repeated within 24 h and between the third to fifth day from admission, in 1 study within 5 days from admission and 1 year after and in 1 study blood samples were obtained consecutively every two days from day 1 to day 13.

### 3.6. Scales of Stroke Severity and Prognosis/Clinical Outcome

NIHSS and the modified Rankin scale (mRS) were used solely in 1 study each. NIHSS and mRS were simultaneously used in 12 studies. In 1 study, the modified NIHSS (mNIHSS) and the Barthel Index (BI) were combined, In 2 studies, the intracerebral hemorrhage (ICH) score and mRS were used. 2 studies assessed both the WFNS grade and the Glasgow outcome scale (GOS), one study graded WFNS and mRS and finally, in 1 study, the modified ICH score (MICH) and mRS were utilized.

**Table 1:** Characteristics of the 25 included studies.

	Authors, Year of Publication	Type of Study	Number of Participants /Mean or Median Age	Time of Copeptin Measurement	Follow-Up Time	Assessment Stroke Scales	Cutoff Values; (Specificity); [Sensitivity]	Main Results
Ischemic Stroke (IS)								
1.	DeMarchis et al., 2013 [27]	Longitudinal	783 patients/median age: 71 (60.5–80)	Within 24 h from symptom onset	3 months	NIHSS (on admission) mRS (at 3 months)		Copeptin may act as an independent predictor of both unfavorable functional outcome and mortality at three months following stroke, as well as accurately forecast the development of in-hospital complications, providing additional valuable prognostic information
2.	Perovic et al., 2017 [28]	Longitudinal	109 patients/median age: 78 (69–84), 63 controls/median age: 75 (70–77)	Within 24 h frοm symptom onset	At discharge (median in-hospital stay 10 days)	mNIHSS (on admission) BI (at discharge)		Copeptin concentrations early after stroke onset were negatively correlated with functional outcome at discharge
3.	Tu et al., 2017 [34]	Longitudinal	4215 patients	Within 48 h from symptom onset	1 month, 6 months, 1 year	NIHSS (on admission)		Elevated plasma copeptin levels were strongly associated with the group of non-survivors, supporting the utility of copeptin as an independent indicator of stroke-related mortality
4.	Wang et al., 2016 [37]	Longitudinal	247 patients/median age: 65 (54–77)	Within 48 h from symptom onset	3 months	NIHSS (on admission) mRS (at 3 months)	For unfavorable functional outcome: 15.4 pmol/L; (84.6%); [62.8%]	Baseline copeptin levels were found to be strongly correlated not only with unfavorable functional outcome but also with mortality, independently of NIHSS and other known risk factors in IS patients diagnosed with type 2 diabetes mellitus
5.	Zhang et al., 2013 [35]	Longitudinal	245 patients/mean age: 72 ± 11, 100 controls	Within 72 h from symptom onset	1 year	NIHSS (on admission) mRS (at 1 year)	For 1 year mortality: 12.55 pM	Significantly higher copeptin levels on admission were detected among patients with poor functional outcomes and non-survivors following an IS. Copeptin evaluation may increase the prognostic ability of the established clinical score
6.	Dong et al., 2013 [29]	Longitudinal	125 patients/median age: 69 (61–85), 100 controls	Within 48 h from symptom onset	3 months	NIHSS (on admission) mRS (at 3 months)		Elevated baseline copeptin concentrations were coupled with increased severity of stroke and were accompanied by both an unfavorable functional outcome and higher mortality risk at 3 months poststroke
7.	Hotter et al., 2020 [38]	Longitudinal	573 patients/mean age: 72.1 ± 12.2	Within the first 4 days of admission	3 months	NIHSS (on admission) mRS (at 3 months)	For SAP: 6.2 μg/L; (30%); [96%]	Copeptin has the potential to independently predict the development of pneumonia during hospitalization, as well as reliably provide an estimation of the functional outcome at 3- months poststroke. However, the added prognostic value of copeptin was found to be limited, while no correlation was demonstrated between plasma copeptin level and mortality
8.	Spagnolello et al., 2019 [30]	Longitudinal	34 patients/mean age: 70.5 ± 16.8	at baseline at 24 h between third and fifth day from admission	1 year	NIHSS (on admission) mRS (at 1 year)		Plasma copeptin levels at 24 h were strongly correlated with poor outcome and mortality at 1-year poststroke, potentially related to brain edema or hemorrhagic transformation. The copeptin’s decremental course within 24 h poststroke was found significantly steeper in patients undergoing combined recanalization strategies
9.	Wang et al., 2014 [36]	Longitudinal	285 patients/median age: 68 (60–79), 100 controls/median age: 68 9 (60–79)	On the first day of admission	1 year	NIHSS (on admission) mRS (at 1 year)	For mortality: 20.5 pmol/L; (84.5%); [90.7%]	Copeptin measurement might add valuable predictive information beyond stroke severity and reliably forecast 1- year mortality in patients presenting with IS
10.	Tu et al., 2013 [31]	Longitudinal	189 patients/median age: 66 (58–75), 200 controls	Within 48 h from symptom onset	3 months	NIHSS (on admission) mRS (at 3 months)		Early measurement of plasma copeptin levels may serve as an independent prognostic outcome predictor with the greatest prognostic potential among the biomarkers under research. A biomarker panel including copeptin might accurately predict unfavorable outcome at 90 days poststroke
11.	Hotter et al., 2019 [33]	Longitudinal	91 patients/mean age: 68.0 ± 10.5	Within the first 4 days of admission	3 months	NIHSS (on admission) mRS (at 3 months)		Copeptin evaluation was significantly associated with functional outcome at 90 days poststroke, thus ultrasensitive copeptin may add useful prognostic information after stroke
12.	Oraby et al., 2021 [32]	Longitudinal	45 patients/mean age: 55.2 ± 13.8, 45 controls/mean age: 51.13 ± 13.4	Within 24 h from symptom onset	3 months	NIHSS (on admission) mRS (at 3 months)	For unfavorable outcome: 125.30 pg/mL; (84.4%); [62.2%]	Elevated copeptin levels were highly correlated with a more severe stroke, as well as poor short-term functional outcome at 3 months. Lower copeptin concentrations were found in the group of patients undergoing thrombolytic therapies
**Transient ischemic attack (TIA)**								
13.	Pedersen et al., 2019 [42]	Longitudinal	114 patients/median age: 66.3 (54.5–71.9)	Within 24 h from symptom onset	Median cardiac monitoring time: 2.2 years	N/A		Copeptin was of limited value in forecasting AF among TIA patients
14.	De Marchis et al., 2014 [41]	Longitudinal	302 patients/median age: 69 (59–78)	Within 24 h from symptom onset	3 months	N/A	For stroke after TIA: 1.88 pmol/L; (12%); [100%] 53.50 pmol/L; (90%); [27%]	Plasma baseline copeptin levels were strongly correlated with recurrent stroke but not TIA within 3 months after the index TIA. Copeptin assessment seems to improve the discriminatory accuracy of ABCD2 score
15.	Purroy et al., 2016 [40]	Longitudinal	237 patients	Within 24 h from symptom onset	7 days, 3 months	mRS (at baseline)	For stroke recurrence: 13.8 pmol/L had a great negative prognostic value (97.4%). Prognostic accuracy was 66.7%.	Abnormally high copeptin concentrations 24 h after TIA symptom onset appears to be indicative of recurrent stroke at 7 days follow-up, but not at 3 months
16.	Griesenegger et al., 2015 [39]	Longitudinal	1076 patients/median age: 75 (66–83), 401 controls	within 5 days from symptom onset at 1 year	Median follow up time: 5, 7 years	N/A		In patients with TIA and ischemic stroke, copeptin was highly predictive of recurrent vascular events and death, especially after TIA or stroke of cardioembolic source
**Intracerebral hemorrhage (ICH)**								
17.	Yu et al., 2014 [43]	Longitudinal	118 patients/mean age: 64.1 ± 9.1, 118 controls/mean age: 62.3 ± 7.8	Within 6 h from symptom onset	6 months	NIHSS (on admission) mRS (at 6 months)	For mortality: 2518.2 pg/mL; (74.1%); [78.4%] For unfavorable outcome: 2369.1 pg/mL; (82.0%); [70.6%]	Significantly higher copeptin concentrations were found on admission among non-survivors and patients with poor functional outcome within 6 months following ICH. Only copeptin has the potential to improve the predictive performance of NIHSS scale
18.	Zhang et al., 2013 [45]	Longitudinal	120 patients/mean age: 60 ± 14, 60 controls	On admission	3 months	ICH Score (on admission) mRS (at 3 months)		Elevated copeptin concentrations were observed among ICH patients with impaired nerve function and unfavorable functional outcome at 90 days following hemorrhage
19.	Zhang et al., 2012 [44]	Longitudinal	89 patients/mean age: 64.5 ± 10.9, 50 controls	On admission	1 year	NIHSS (on admission) mRS (at 1 year)	For mortality: >23.8 pmol/L; (70.6%); [81.6%] For unfavourable outcome: >23.5 pmol/L; (87.9%); [76.8%] For END: >26.3 pmol/L; (73.1%); [81.8]	Increased plasma copeptin level may serve as an independent prognostic marker of 1- year mortality, 1-year unfavorable outcome, and early neurological deterioration after ICH, but it does not improve significantly the predictive value of NIHSS score
20.	Yang et al., 2021 [47]	Longitudinal	156 patients/mean age: 45.06 ± 9.78	Within 24 h of admission	3 months	MICH score (on admission) mRS (at 3 months)		Baseline plasma copeptin levels were markedly higher within the non- survivors group accompanied by the copeptin concentrations among the ICH patients with poor functional outcome at 3-month follow up
21.	Wei et al., 2014 [46]	Longitudinal	271 patients/median age: 69 (59–81), 200 healthy controls/ median age: 69 (58–80)	Within 48 h from symptom onset	3 months	ICH score (on admission) mRS (at 3 months)		Increased copeptin levels were found within ICH population with poor prognosis and non- survivors, suggesting the role of copeptin as an independent marker of functional outcome and death at 3-month follow- up
**Subarachnoid hemorrhage (SAH)**								
22.	Fung et al., 2013 [48]	Longitudinal	18 patients/median age: 57 (48–67)	On admission	6 months	WFNS (on admission) mRS (at 6 months)		Circulating copeptin levels were found to be strongly correlated with SAH severity, as assessed by the WFNS scale. Copeptin seems to have an interesting prognostic potential regarding functional outcomes at 6 months, as it tended to be higher among patients with poor prognosis
23.	Zuo et al., 2019 [50]	Longitudinal	243 patients/median age: 58 (49–69)	Within 48 h from symptom onset	3 months	WFNS (on admission) Glasgow outcome scale (at 3 months)	For poor outcome: 24.0 pmol/L; (69.6%); [70.5%]	Copeptin evaluation may serve as an independent marker of short-term prognosis after SAH, with elevated copeptin concentrations being detected among non-survivors and SAH patients with poor functional outcome at 3 months. The prognostic accuracy was in the range of WFNS scale
24.	Rhim et al., 2021 [51]	Longitudinal	86 patients	Consecutive measurements every 2 days from day 1 until day 13	13 days	N/A		Elevated copeptin concentrations stand for a significant risk factor for delayed cerebral ischemia (DCI) occurrence throughout SAH clinical course, enabling a better risk stratification for SAH patients
25.	Zheng et al., 2017 [49]	Longitudinal	105 patients/median age: 52 (37–60)	On admission	6 months	WFNS (on admission) Glasgow Outcome Scale (at 6 months)		Copeptin levels were associated with WFNS scale scores, reflecting SAH severity. SAH patients with an unfavorable 6-month clinical outcome, as well as patients developing symptomatic cerebral vasospasm carried higher copeptin levels on admission

Abbreviations: IS: ischemic stroke, NIHSS: National Institutes of Health Stroke Scale, mRS: modified Rankin scale, mNIHSS: modified National Institutes of Health Stroke Scale, TIA: transient ischemic attack, AF: atrial fibrillation, ICH: intracerebral hemorrhage, MICH score: modified Intracerebral hemorrhage score, SAH: subarachnoid hemorrhage, WFNS score: World Federation of Neurological Surgeons score, N/A: not applicable.

## 4. Discussion

A literature review over the last decade was conducted in order to elucidate the prognostic value of copeptin after stroke. Twenty-five full-text original articles dealing with the potential utility of the evaluation of copeptin plasma levels on stroke prognosis were identified and classified into groups based on the stroke subtype under investigation (Table 1).

### 4.1. Ischemic Stroke

With respect to acutely ischemic stroke patients, De Marchis et al. [27] suggest that copeptin has an important prognostic value, as the aforementioned blood biomarker appears to serve as an independent predictor of both unfavorable functional outcome and mortality at three months following stroke, as well as reliably forecast the development of in-hospital complications. Interestingly, copeptin has the potential to enhance the discriminatory accuracy of clinical variables independent of the type of acute treatment offered (conservative vs. recanalization therapy). The combined assessment of copeptin blood levels with a validated prognostic score incorporating both NIHSS and age significantly improves risk stratification regarding functional outcome and mortality poststroke. Additionally, Perovic et al. [28], in an attempt to explore the possible predictive role of copeptin on short-term outcome, studied 112 patients with AIS and found that copeptin levels were higher in patients with lower Barthel Index (BI) scores. Thus, a significant negative correlation between copeptin concentration early after stroke onset and functional outcome at discharge was revealed.

Apart from that, Dong et al. [29], having enrolled 125 patients with AIS, investigated the potential utility of copeptin as a prognostic tool for outcome and mortality during a 90-day follow-up from stroke onset. The researchers concluded that elevated baseline copeptin concentrations were coupled with increased severity of stroke and were accompanied by both an unfavorable functional outcome and higher mortality risk. Thus, higher plasma copeptin levels detected in stroke patients with poor outcomes and nonsurvivors may significantly enhance the identification of patients at highest risk for adverse outcomes. As far as poststroke copeptin kinetics are concerned, Spagnolello et al. [30] explored the temporal profile of copeptin in relation to the application of reperfusion therapeutic strategies, as well as the development of subsequent complications after an acute ischemic stroke. They reported similar findings with preexisting data, as they confirmed the positive association between plasma copeptin concentration on admission and at 24 h following stroke and stroke severity and unfavorable functional outcomes at 1 year, respectively. Importantly, the aforementioned researchers determined for the first time the temporal relationship between circulating copeptin levels and the occurrence of stroke-related brain edema and hemorrhagic transformation. It is of great interest that copeptin decremental course within 24 h poststroke was found to be significantly steep in patients undergoing combined recanalization strategies, thus potentially reflecting a successful revascularization effect. On the contrary, patients receiving either thrombolysis/thrombectomy alone or conservative therapy exhibited a less rapid decrease of blood copeptin levels.

Regarding copeptin as a candidate for inclusion in a biomarker panel aimed at prognosticating short-term stroke outcomes, Tu et al. [31] studied 189 patients after an acute ischemic stroke and observed that the early measurement of plasma copeptin levels may serve as an independent prognostic outcome predictor with the greatest prognostic potential among the biomarkers under research. It is noteworthy that the evaluation of copeptin concentration within a biomarker panel is found to be coupled with greater prognostic potential following stroke than the assessment of a single blood biomarker. Moreover, in a study conducted by Oraby et al. [32] it was found that elevated copeptin levels were strongly correlated with a more severe stroke, as well as poor short-term functional outcome at 3 months. In agreement with aforementioned data, the researchers reported lower plasma copeptin concentrations in the group of patients undergoing thrombolytic therapies compared to those that were not eligible for reperfusion interventions. Similarly, Hotter et al. [33], in an attempt to investigate the relationship between blood biomarkers and stroke outcome, concluded that ultrasensitive copeptin may add valuable prognostic information following stroke, as its evaluation was significantly associated with functional outcome at 90 days poststroke.

As far as the long-term mortality following an acute ischemic stroke is concerned, Tu et al. [34], having examined 4125 stroke patients, demonstrated the prognostic potential of copeptin in predicting all-cause death or cardiovascular disease (CVD) mortality during a follow-up period of 1-year poststroke. The researchers support the utility of copeptin as an independent prognostic indicator of stroke-related mortality, as elevated plasma copeptin levels were strongly associated with the group of nonsurvivors in contrast to the copeptin concentrations within the survivors group. Similarly, Zhang et al. [35] reported significantly higher copeptin levels on admission among patients with poor functional outcomes and non-survivors following an AIS, emphasizing the predictive significance of copeptin regarding long-term outcome and mortality. Importantly, copeptin appears to closely reflect stroke severity by being positively correlated with NIHSS, while increasing the prognostic ability of the established clinical score. Moreover, in a study conducted by Wang et al. [36], it was observed that copeptin measurement might add valuable predictive information beyond stroke severity (assessed by the NIHSS) and reliably forecast 1-year mortality in patients presenting with AIS. It is noteworthy that copeptin levels seem to parallel both with lesion size and neurological deficit poststroke.

With regard to the prognostic utility of copeptin measurement within specific stroke subpopulations, Wang et al. [37] enrolled 247 stroke patients with type 2 diabetes mellitus in an attempt to provide insight into the potential linkage between copeptin levels at admission and short-term functional outcome following an acute ischemic stroke in patients diagnosed with type 2 diabetes mellitus. The researchers reported a positive correlation between copeptin plasma concentration and both stroke severity as defined by the NIHSS score and lesion size as assessed by MRI. At 3-month follow-up, baseline copeptin levels were found to be strongly correlated not only with unfavorable functional outcome but also with mortality, showing a significantly higher discriminatory accuracy in predicting poor outcomes when compared to blood hemoglobin a1c (HbA1c) measures. Interestingly, it was demonstrated that copeptin has the potential to forecast the development of unfavorable functional outcome poststroke independently of NIHSS and other known risk factors.

In contrast to the aforementioned research data, Hotter et al. [38] attempted to investigate the prognostic ability of copeptin in a stroke setting regarding the short-term functional outcome, death, and stroke-related complications, especially pneumonia. According to the study’s findings, copeptin appears able to independently predict the development of pneumonia during hospitalization, as well as reliably provide an estimation of the functional outcome at 3-months poststroke. However, the added prognostic value of the blood biomarker is found to be limited, only slightly improving the overall predictive ability of clinical variables regarding stroke-related pneumonia and functional outcome following stroke. Moreover, death at 3 months poststroke was accurately predicted by clinical parameters including age and baseline NIHSS score, while no correlation was found between plasma copeptin level and mortality.

### 4.2. Transient Ischemic Attack

Regarding the role of copeptin as a prognostic blood biomarker following a TIA, Greisenegger et al. [39] having enrolled 1076 patients presented with ischemic stroke or TIA observed that copeptin has the potential not only to forecast the long-term risk of recurrent vascular events and ischemic stroke but also to be predictive of vascular and all-cause death after TIA or stroke. Interestingly, the prognostic value of copeptin for re-events was significantly higher among patients with stroke or TIA of cardioembolic source, as elevated plasma copeptin levels within this group were accompanied by a 4-fold increased risk of subsequent vascular events during 1-year follow-up. Additionally, the researchers observed that copeptin measurement seems to provide valuable prognostic information beyond established clinical variables, thus facilitating patients’ early risk stratification and guiding decision-making post-stroke/TIA. Similarly, Purroy et al. [40], in an attempt to provide insight into the predictive ability of a biomarker-based approach among TIA patients, reported significantly higher copeptin levels in patients with stroke recurrence within 7 days but not within 3 months following TIA. Thus, abnormally high copeptin concentration 24 h after TIA symptom onset appears to be indicative of recurrent stroke at 7 days follow-up, enhancing risk classification and overall management of TIA patients.

As far as the incremental value of copeptin compared with ABCD2 score is concerned, De Marchis et al. [41], having studied 302 TIA patients, demonstrated that plasma baseline copeptin levels were strongly correlated with recurrent stroke within 3 months after TIA, while similar association between copeptin concentration and TIA recurrence during follow-up was not observed. Of note, copeptin assessment improved the discriminatory accuracy of ABCD2 score, differentiating between TIA patients at high versus low stroke risk and enabled a more accurate risk stratification after TIA. Apart from that, Pedersen et al. [42] aimed to explore the role of copeptin as a marker of atrial fibrillation (AF) in TIA patients evaluated 114 patients after TIA and extensive cardiac monitoring in order to identify potential AF indicators. The researchers concluded that copeptin, among other blood biomarkers, was of limited value in forecasting AF among TIA patients.

### 4.3. Intracerebral Hemorrhage

With regard to the prognostic utility of copeptin in an acute ICH setting, Yu et al. [43], having enrolled 118 ICH patients, investigated the predictive performance of copeptin in comparison with other damage blood biomarkers. Additionally, this study also demonstrated a strong correlation between plasma copeptin levels and both long-term mortality and unfavorable functional outcome after ICH. More specifically, the researchers reported significantly higher copeptin concentrations on admission among nonsurvivors and patients with poor functional outcome within 6 months following ICH than copeptin levels in ICH patients who survived and exhibited a favorable functional outcome. Although copeptin and the other explored biomarkers were found to be characterized by a predictive ability similar to that of NIHSS score, only copeptin has the potential to significantly improve the discriminatory accuracy of NIHSS score regarding 6-month unfavorable functional outcome, highlighting the potentially valuable role of copeptin in clinical practice. Similarly, Zhang et al. [44], in an attempt to provide further insight into the relationship between early copeptin evaluation and clinical outcome among ICH patients, studied a group of 89 patients. Their research has shown that plasma copeptin levels were significantly associated with long-term functional outcome and mortality, thus representing an independent prognostic indicator during 1-year follow-up after ICH. Regarding early neurological deterioration (END), it was reported that elevated copeptin concentration was found to be indicative of END in acute ICH and as a result, copeptin measurement on admission may discriminate patients at high- versus low-END risk. Moreover, the predictive ability of copeptin was reported to be in the range of NIHSS score, but unlike the Yu et al. [43] findings, the copeptin assessment did not significantly enhance the NIHSS prognostic value.

Furthermore, in a study that was conducted by Zhang et al. [45] and included 120 ICH patients, it was demonstrated that admission copeptin levels were positively correlated with hematoma volume and negatively associated with GCS, thus reflecting the clinical severity of cerebral hemorrhage. The researchers reported elevated copeptin concentrations among ICH patients with impaired nerve function and unfavorable functional outcome at 90 days following hemorrhage, indicating the copeptin’s role as an independent predictor of 3-month clinical outcome. Regarding mortality prediction, even though it was not statistically significant, plasma copeptin level was found to be higher in the group of nonsurvivors than in the survivors. In agreement with previous findings, Wei et al. [46] showed that plasma copeptin concentration on admission was correlated with initial hematoma volume, which in turn stands for a severity and outcome indicator. Having studied 271 ICH patients, the researchers concluded that copeptin may serve as an independent powerful tool to forecast both functional outcome and death 90 days after ICH, as increased copeptin levels were found within ICH population with poor prognosis and nonsurvivors.

Apart from that, Yang et al. [47] attempted to explore the discriminative power of copeptin in conjunction with glial fibrillary acidic protein (GFAP), C-reactive protein (CRP) and clinical assessment tools, as the modified intracerebral hemorrhage rating score (MICH), in patients with ICH. According to the study’s findings, the baseline plasma copeptin levels were markedly higher within the nonsurvivors group accompanied by the copeptin concentrations among the ICH patients with poor functional outcome at 3-month follow up. Both the unfavorable prognosis group and the death group were characterized by significantly increased copeptin levels when compared to the good prognosis group after ICH. Importantly, copeptin level on admission seems to parallel the clinical evaluation score MICH, thus representing an accurate measure of ICH severity. The researchers concluded that the combined implementation of serum copeptin, GFAP and CRP levels with clinical parameters in an ICH setting might considerably enhance the evaluation of prognosis for ICH patients.

### 4.4. Subarachnoid Hemorrhage

As far as the prognosis following SAH is concerned, Fung et al. [48] having studied a population of 18 patients presented with aneurysmal subarachnoid hemorrhage (aSAH) explored the potential prognostic significance of plasma copeptin on admission regarding severity and clinical outcome. They reported a strong correlation between baseline copeptin concentration and severity of aSAH as assessed by World Federation of Neurological Surgeons (WFNS) score, as well as between copeptin levels and both the amount of subarachnoid blood and the occurrence of ICH among aSAH patients. The association of circulating copeptin levels with the gold standard evaluation tool for SAH, WFNS grade, may act as an indicator of initial bleeding severity, thus significantly facilitating the management in an acute SAH setting. Additionally, it was found that copeptin concentration tended to be higher within the poor prognosis group of patients, exhibiting a prognostic potential for functional outcome at 6 months after SAH.

In a study conducted by Zheng et al. [49], it was demonstrated that plasma copeptin concentrations were significantly associated with WFNS scale scores in SAH patients, reflecting the severity of the bleeding event. Moreover, it was observed that SAH patients with an unfavorable 6-month clinical outcome, as well as patients developing symptomatic cerebral vasospasm, carried higher copeptin levels on admission when compared to those with good prognosis and without symptomatic cerebral vasospasm, respectively. Comparing copeptin with both other acute brain injury markers and clinical WFNS score in terms of predictive ability, the researchers showed that the prognostic value of all the investigated biomarkers was in the range of baseline WFNS, but only copeptin was able to enhance the predictive power of WFNS grade as far as the prognostication of poor functional outcome and cerebral vasospasm is concerned. Zuo and Ji [50] further confirmed the aforementioned findings regarding the superiority of copeptin in predicting SAH outcome compared to other commonly utilized biomarkers. The researchers demonstrated that an increase in plasma copeptin levels was accompanied by an increase in SAH severity measured by the WFNS score, with a similar prognostic accuracy between copeptin and clinical WFNS scale. Furthermore, the copeptin evaluation was found to be able to serve as an independent marker of short-term prognosis after SAH, with elevated copeptin concentrations being detected among nonsurvivors and SAH patients with poor functional outcome at 3 months. Apart from that, Rhim et al. [51], having examined 86 patients, explored the relationship between copeptin levels and the development of delayed cerebral ischemia (DCI) in patients with poor grade SAH. According to the study’s findings, elevated copeptin concentrations stand for a significant risk factor for DCI occurrence throughout SAH clinical course, as higher copeptin levels were found in the DCI group than in the non-DCI group. The discriminatory ability of copeptin regarding DCI might enable better risk stratification among SAH patients.

### 4.5. Study Limitations

Some limitations of the included studies in the present review merit attention and must be carefully considered. First, most studies reported a relatively small sample size, as well as a single-center design, that may adversely affect the statistical power of the observations and potentially limit the generalization of the results. Second, the evaluation of all-cause mortality, due to the complexity of death classification within a clinical setting, might impact the causal link between stroke and death events. Third, a single measurement of serum copeptin concentration following stroke, as performed by many of the included studies, appears not able to provide additional information regarding the onset and duration of circulating copeptin levels elevation post-stroke. Fourth, the close relationship of copeptin release with the body’s stress responses and the potential increase of plasma copeptin levels in the setting of medical comorbidity may interfere with the prognostic utility of copeptin among hospitalized stroke patients. Taking the aforementioned limitations into account, there is an emerging need for additional multicenter larger-scale studies performing serial copeptin testing following stroke, in order to investigate whether copeptin evaluation further enhances the risk stratification of stroke survivors. In this direction, it would be ideal to consider comorbidities that may result in copeptin levels increasing independently of a stroke event, as well as to assess data on mortality from vascular events solely, in order to elucidate the direct linkage of copeptin levels with stroke outcome. Moreover, future meta-analysis and mega-analysis studies may further examine the impact of copeptin on stroke, further attempting to facilitate the formation of guidelines for the best course management of stroke patients in several settings based on copeptin evaluation.

## 5. Conclusions

Taking everything into account, the present review provides an overview of the potential clinical applications of blood-derived copeptin as a prognostic biomarker in an acute stroke setting. Copeptin, reflecting the body’s stress response early after a stroke event, may act as a useful prognostic tool, especially when consecutive measurements take place during hospitalization. Our findings support the beneficial use of plasma copeptin measurement in prognostication following stroke, thus indicating that a biomarker-based approach with the evaluation of copeptin may provide important insight into the recovery potential of each stroke survivor and significantly facilitate individualized stroke care. Copeptin seems able to serve as a surrogate marker of stroke severity, as well as differentiate reliably between patients with good prognoses and patients with unfavorable functional outcome. Interestingly, circulating copeptin levels might independently predict both clinical outcome and mortality, as higher copeptin concentrations were mostly found to be coupled with poor prognosis after stroke. Of note, plasma copeptin levels may be also indicative of many complications through stroke clinical course, as pneumonia, early neurological deterioration or delayed cerebral ischemia. It is of great importance that the aforementioned data were not limited to cases of acute ischemic stroke, but similar findings were reported among patients with TIA, ICH, and SAH. Given the fact that copeptin assessment is able to add valuable predictive information beyond clinical variables, it could significantly enhance the discriminatory accuracy of widely utilized validated prognostic scores, thus optimizing overall stroke management. Additional larger-scale well-designed studies among stroke patients on the association between copeptin level and propensity for recovery are recommended in order to further elucidate this clinically important relationship.

## Data Availability

All data discussed within this manuscript is available on PubMed.
